# Wnt signaling induces epithelial differentiation during cutaneous wound healing

**DOI:** 10.1186/1471-2121-7-4

**Published:** 2006-01-20

**Authors:** Carrie Fathke, Lynne Wilson, Kavita Shah, Brian Kim, Anne Hocking, Randall Moon, Frank Isik

**Affiliations:** 1 Department of Surgery, University of Washington School of Medicine, Seattle, WA 98195, USA; 2 Howard Hughes Medical Institute/Department of Pharmacology, University of Washington School of Medicine, Seattle, WA 98195, USA

## Abstract

**Background:**

Cutaneous wound repair in adult mammals does not regenerate the original epithelial architecture and results in altered skin function. We propose that lack of regeneration may be due to the absence of appropriate molecular signals to promote regeneration. In this study, we investigated the regulation of Wnt signaling during cutaneous wound healing and the consequence of activating either the beta-catenin-dependent or beta-catenin-independent Wnt signaling on epidermal architecture during wound repair.

**Results:**

We determined that the expression of Wnt ligands that typically signal via the beta-catenin-independent pathway is up-regulated in the wound while the beta-catenin-dependent Wnt signaling is activated in the hair follicles adjacent to the wound edge. Ectopic activation of beta-catenin-dependent Wnt signaling with lithium chloride in the wound resulted in epithelial cysts and occasional rudimentary hair follicle structures within the epidermis. In contrast, forced expression of Wnt-5a in the deeper wound induced changes in the interfollicular epithelium mimicking regeneration, including formation of epithelia-lined cysts in the wound dermis, rudimentary hair follicles and sebaceous glands, without formation of tumors.

**Conclusion:**

These findings suggest that adult interfollicular epithelium is capable of responding to Wnt morphogenic signals necessary for restoring epithelial tissue patterning in the skin during wound repair.

## Background

Mammalian skin serves a number of vital physiological functions to maintain homeostasis. Skin provides a moisture barrier, regulates body temperature via hair follicles, sweat glands, and dermal capillaries, and provides lubrication via sebaceous glands. The functional properties of skin are often underappreciated until substantial loss of the skin occurs.

Cutaneous repair in adult mammals following full-thickness skin loss results in scar tissue: a collagen-rich dermal matrix with a simple stratified epithelial covering different from the original skin in appearance and function. Deposition of a collagen-rich matrix in the neo-dermis is prone to contracture, loss in elasticity, tensile strength and hypertrophic scar formation. Epithelialization without epidermal appendage development over a large surface area leads to alopecia, desiccation and thermal dysregulation. The underlying problem is that cutaneous wounds in the adult mammal do not heal by regeneration of the original tissue architecture [1].

Regeneration is not observed during adult cutaneous wound healing despite the presence of multipotent epidermal stem cells in the hair follicle bulge [2,3] and undifferentiated mesenchymal cells in the dermis [4,5]. The existence of undifferentiated cells in the skin suggests that skin has the potential to regenerate, but the context of molecular signals after tissue injury promotes scar repair, not regeneration. We hypothesized that the lack of cutaneous regeneration following wounding results from the absence of molecular signals that guide tissue patterning for restoration of the original skin architecture. In this study, we examined the consequence of activating Wnt signaling during cutaneous wound healing.

Members of the Wnt family are secreted glycoproteins that regulate cell proliferation, migration and specification of cell fate in the embryo and adult [6]. Wnt proteins are classified according to their ability to promote stabilization of β-catenin in the cytoplasm. The β-catenin-dependent Wnt pathway signals through cytoplasmic stabilization and accumulation of β-catenin in the nucleus to activate gene transcription. In contrast, a number of alternative signaling mechanisms including calcium flux, JNK and heterotrimeric G-proteins have been implicated in β-catenin-independent Wnt signaling (reviewed in Veeman *et al*. [6]).

There is increasing evidence that Wnts are necessary for normal skin development (for review, see [7]). β-catenin-dependent signaling has been shown to be involved in hair follicle morphogenesis. Expression of stabilized β-catenin in the epidermis of transgenic mice resulted in hair follicle morphogenesis [8]. The hair follicles formed complete with sebaceous glands and dermal papilla, but ultimately led to hair follicle tumors. Conversely, when β-catenin expression was ablated in the epidermis, hair follicle morphogenesis was blocked [9]. This study also revealed that β-catenin has an important role in specifying the cell fate of skin stem cells, where absence of β-catenin favored differentiation into epidermal rather than follicular keratinocytes.

In contrast, the function of β-catenin-independent Wnts such as Wnt-4, Wnt-5a and Wnt-11 in normal skin is unknown; however, we emphasize that these Wnts may also activate the β-catenin-dependent pathway depending on the cellular context. Wnt-4 is expressed in the epidermis of both embryonic and adult mouse skin and Wnt-5a and Wnt-11 are expressed in the dermis of embryonic mouse skin [10]. Although correlative data suggests that Wnt-5a may be a downstream target of sonic hedgehog involved in hair follicle morphogenesis, the function of Wnt-5a and the role of β-catenin-independent Wnt signaling in skin remain unclear.

The aim of this study was to determine the regulation of Wnt expression during adult mammalian wound repair and to investigate whether activation of either β-catenin-dependent or β-catenin-independent Wnt signaling could result in regenerative changes in the skin during wound healing. We observed that the expression levels of the β-catenin-independent Wnts, Wnt-4, -5a and -11 were up-regulated transiently during cutaneous wound healing. In addition, we observed a similarly transient activation of the β-catenin-dependent Wnt pathway, but limited to the epithelial hair follicles adjacent to the wound; not within the wound or overlying epithelium. We found that the prolonged activation of the β-catenin-dependent pathway resulted in epithelial appendages forming in the wound, including epithelial cysts within the dermis, hair follicles and occasional sebocytes, features not seen in the control group. Retroviral expression of Wnt-5a in the dermal wound resulted in similar but more abundant epithelial appendage formation including epithelial-lined cysts, rudimentary hair follicles and sebaceous glands, without the formation of tumors. In summary, our data suggests that the prolonged activation of β-catenin-dependent Wnt signaling by lithium chloride or the prolonged expression of Wnt-5a promotes partial regeneration of epithelial appendages in adult murine skin and demonstrates the plasticity of adult epithelial cells during wound healing.

## Results

### Wnt expression in murine skin and wound repair

**Figure 1:**
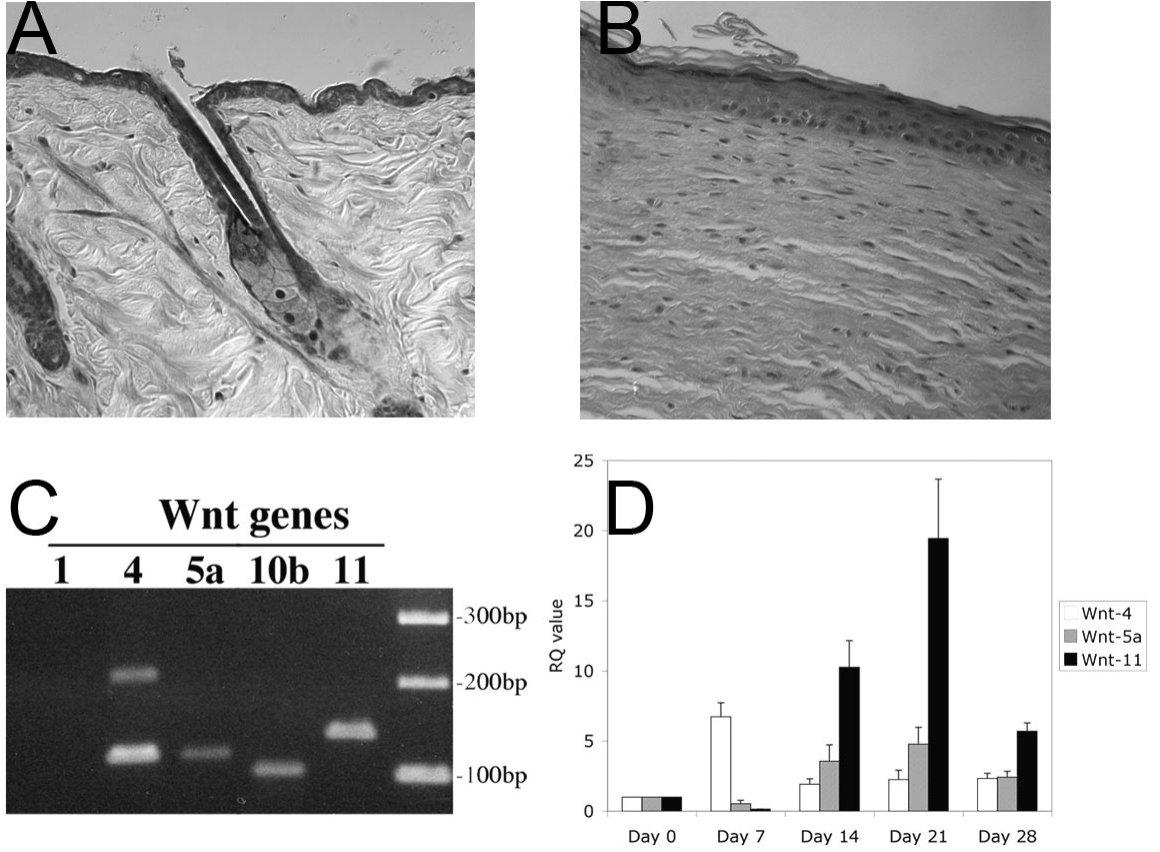
(a) Representative normal murine skin shows the presence of an orderly collagen in the dermis and hair follicles, with associated sebacecous glands. (b) After a full-thickness wound has healed, the skin forms a stratified epithelium over a fibroblast-rich dermis, lacking the epithelial appendages seen in 1a. (c) PCR analysis of normal murine skin shows the presence of Wnt-4, -5a, -7a, -10b and -11. (d) Real time PCR analysis of wounds examined at several time points shows Wnt-4 expression peaking at day 7 after wounding, whereas Wnt 5a and -11 expression levels peak 21 days after wounding, or 1 week after epithelialization has completed.

In the C57BL mouse wound healing model, a 1.5 cm^2 ^dorsal wound represents a relatively large surface area that will heal mostly by contraction and limited epithelialization by day 14. Circumferential contraction of the wound edges draws the normal surrounding skin towards the center of the wound. The remaining portion of the wound heals by epithelialization with a simple stratified interfollicular epithelium, but lacks epithelial appendages such as hair follicles or sebaceous glands (Figs. 1a &1b).

Endogenous Wnt mRNA expression in response to cutaneous injury was examined in BATGAL mice using real time PCR. RNA was extracted from normal skin and wounds harvested on days 0 (normal skin), 7 (inflamed open wound), 14 (recently epithelialized and closed wound), 21 and 28 (wound remodeling) after injury. We found several Wnt genes expressed in normal skin, including transcripts for Wnt-4, Wnt-5a, Wnt-7a, Wnt-10b and Wnt-11 (Fig. 1c) in keeping with prior studies [10,11]. We did not detect expression for Wnt-1. When we examined the temporal expression of pre-selected Wnt genes during wound repair, we consistently saw increased expression for three Wnt members: Wnt-4, Wnt-5a and Wnt-11 (Fig. 1d). We observed increased expression of Wnt-4 shortly after injury peaking on day 7 and temporally coinciding with an inflamed open wound. In contrast, Wnt-5a and Wnt-11 expression levels peaked on day 21, one week following wound closure was achieved. The expression levels for Wnt-4, Wnt-5a and Wnt-11 decreased towards normal skin expression values by day 28. These temporal expression data confirm that transiently increased Wnt mRNA expression is part of the response to cutaneous injury and suggest that Wnt signaling maybe active during the re-epithelialization phase of wound healing.

### Activation of the β-catenin-dependent pathway during wound repair

**Figure 2:**
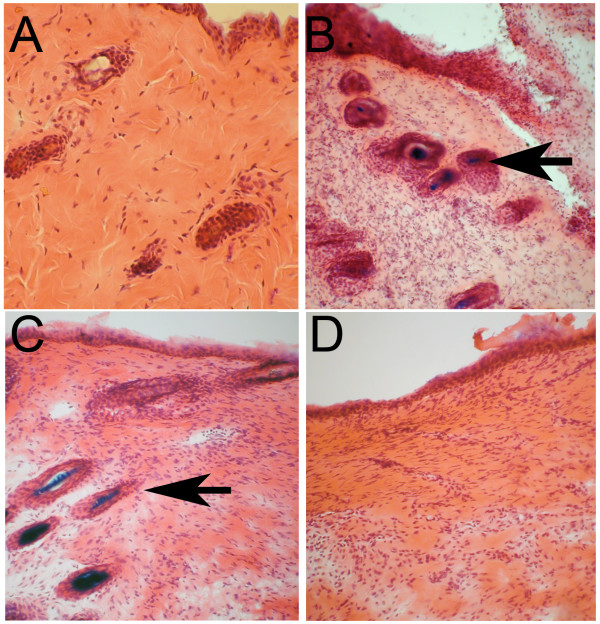
(a) β-galactosidase staining of normal skin from TOPGAL mice, which express the β-galactosidase reporter gene under the control of a promoter responsive to β-catenin-dependent signaling shows little to no β-galactosidase activity. (b) β-galactosidase staining 7 days after wounding shows activity present in the differentiated hair follicle shafts immediately adjacent to the wound edge (arrow). The actual wound is to the right of the arrow. (c) The staining for β-galactosidase was most intense 21 days post wounding, with the wound being to the right of the arrow. (d) At no time point was β-gal activity detected within the wound itself (a representative 21 day wound is shown).

To determine whether β-catenin-dependent Wnt signaling was activated during wound healing, we performed wounding experiments on 8 TOPGAL mice. TOPGAL mice express the β-galactosidase reporter gene under the control of a promoter responsive to β-catenin-dependent signaling [12]. Normal skin showed barely detectable β-galactosidase activity (Fig. 2a) in keeping with other studies [12]. On day 7 after injury, β-gal activity was detected but only in a minority of the differentiated hair shafts immediately adjacent to the wound edge (Fig. 2b). The most robust β-gal activity was detected around day 21, after epithelialization had been complete for one week and again limited to the hair shaft. We did not detect signal in the outer root sheath compartment for any of the time points examined. The β-gal activity remained localized to the wound-adjacent normal skin hair follicles (Fig. 2c). No β-gal activity was observed in the stratified epithelium overlying the wound or within the wound mesenchymal cells in the dermis. We repeated these experiments and saw identical results using BATGAL transgenic mice (n = 8, same time points), which express β-galactosidase under the control of a β-catenin/TCF-responsive promoter [13].

Thus, our results showed that the β-catenin-dependent pathway was active during wound repair but limited to the differentiated hair follicle shafts in the normal uninjured skin adjacent to the wound, not within the wound. In support, we did not detect an increase in the expression of Wnts that signal typically via the β-catenin-dependent pathway, such as Wnt-1. Though our real time PCR analysis of normal and injured skin revealed the expression of several Wnts that typically can activate β-catenin-independent signaling, we were unable to determine whether the β-catenin-independent Wnt signaling pathway was activated during wound healing. Unlike TOPGAL or BATGAL transgenic mice, no promoters loyal to the β-catenin-independent pathway have yet been identified.

### Prolonged activation of β-catenin-dependent pathway

Studies demonstrated the requirement for the β-catenin dependent pathway during hair follicle morphogenesis [8,12], yet no activation of this pathway was observed within the wounds of the TOPGAL or BATGAL mice. To determine whether the epithelial keratinocytes proliferating & migrating over the wound can differentiate into follicular keratinocytes, we attempted to activate the β-catenin dependent pathway in the wound.

Lithium chloride (LiCl) inhibits the activity of glycogen synthase kinase-3β (GSK-3β), allowing the accumulation of free β-catenin and leads to activation of the β-catenin-dependent pathway [14]. We applied topical LiCl (20 mM) to murine wounds until wound closure (14 days) and harvested the wounds on day 28 for histology. The mice in these experiments were age and sex-matched with the control group to control for the natural hair cycle.

**Figure 3:**
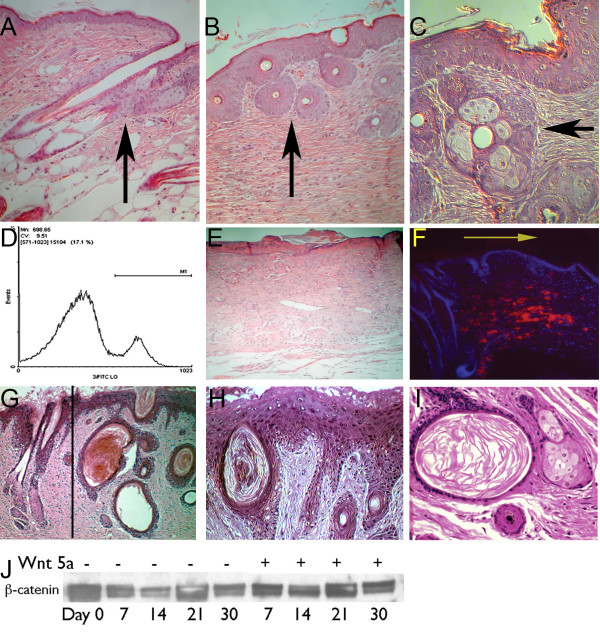
To determine whether Wnt signaling may induce epithelial patterning in wound epithelium as occurs during development, LiCl was applied continuously to the wounds of mice. (a) The adjacent normal skin of a 30 day LiCl treated mouse wound shows normal epithelial appendages and dermal architecture. (b) The same animal, but the wound has been treated with LiCl for 14 days and which shows the presence of epithelial inclusion cysts throughout the wound (arrow; day 30). Note the difference in the dermal architecture between the wounded tissue and normal skin; LiCl treatment had no observable effect on the dermal wound healing response. (c) Occasional larger and more mature appearing epithelial appendages can be seen within the wound (different animal; same time point), demonstrating the formation of sebaceous glands (arrow). Retroviral infected wounds were examined after 21 days and shows CD5 expression in 17% of the wound cells by flow cytometry (d) and (e) shows the histologic effect on the control retroviral treated wound at day 30: lacking any epithelial appendages overlying the wound. (f) Immunofluorescent detection of CD5 shows the infection has occurred in the dermal cells (red staining) adjacent to the normal skin, hair follicles in normal skin can be seen on the left and the wound is to the right of the arrow. By day 30 and persisting through day 90, numerous large epithelia-lined cysts can be seen in the wound (g & h) immediately adjacent to the mature normal skin hair follicles (normal skin is left of the line; wound to the right). (i) These cysts were also associated with sebaceous gland formation. (j) Wounds from PMXWnt-5a treated mice were examined for β-catenin stabilization and compared with control retroviral treated wounds. No significant difference in the amount of free β-catenin was seen between the two groups.

Five out of the 6 mice showed histologic evidence of epithelial appendage formation in their wounds on day 28. As seen in figure 3b, the simple stratified epithelium that typically forms over the wound displayed numerous inclusion cyst-like structures superficially within the epidermis and occasionally, formation of primitive hair follicle structures and sebaceous glands (Fig. 3c). These structures were noted well within the wound, not near or in the adjacent normal skin (Fig. 3a). No changes in the healed dermis with respect to the inflammatory cells, collagen deposition or mesenchymal cells were noted in any of the mice. These findings demonstrate that the adult interfollicular keratinocytes are capable of converting to a follicular phenotype during wound healing, provided the appropriate cell signals are present.

### Prolonged activation of β-catenin-independent pathway

Prior studies had shown that the β-catenin-independent signaling pathway might also play an important role during hair follicle development [8,10]. To determine whether the β-catenin-independent Wnt signaling pathway could influence epithelial tissue patterning during wound healing, we adapted a bicistronic retroviral vector (PMXIRES) containing a non-functional human CD5 epitope as a marker gene and inserted full-length Wnt-5a, which typically signals via the β-catenin-independent pathway. We treated excisional cutaneous wounds with the infectious retrovirus PMXWnt-5a or the control retrovirus containing the CD5 marker alone. Infectivity of the retrovirus was determined by daily application of topical retrovirus-containing media to the wounds for 14 days and on day 21, excised the entire wound and assessed CD5 cell surface marker expression by flow cytometry (Fig. 3d) [15]. Despite multiple variations to our protocol, we achieved only 9–17% infectivity in the wounds. Though low, this seemed to be an acceptable trade-off for stable expression of a secreted protein.

The control retrovirus had no effect on wound histology for any of the time points examined (representative day 90 wound shown in figure 3e) and comparison of untreated mice wounds with control PMXIRES or PMXWnt-5a retrovirus-treated wounds showed no difference in the time to heal (14 days + 1.5 days; data not shown). There was no difference in the degree of epithelialization or wound contraction. Localization of the retroviral infection with PE-conjugated anti-human CD5 antibody showed staining limited to the dermal mesenchymal layer in the wound adjacent to the normal skin, but not in the epidermis (Fig. 3f).

Prior to day 21, no gross changes were noted in the PMXWnt-5a infected wounds. However, the PMXWnt-5a retrovirus treated wounds displayed histologic changes by day 30 that were not seen in the control wounds. Greater than seventy-five percent of the Wnt-5a treated wounds (11/14 mice; Table I) showed evidence of epithelial elements within the deeper dermis on day 30, forming large epithelia-lined cysts with keratinized dead cell layers within the core of the cyst (Figs. 3g &3h). In addition, the wound bed contained small sebaceous glands adjacent to the epithelial cysts and rudimentary hair follicles (Fig. 3i). There were no histologically detected changes in the healed dermis with respect to collagen abundance, collagen orientation, vascularity or inflammation between the experimental and control groups.

**Table 1:** Summary of our results. None of the control mice developed epithelial appendages during normal wound healing. However, the mice treated with topical lithium chloride showed histologic changes in 5 out of 6 wounds. The histologic changes were more evident when the infectious retrovirus containing Wnt5A was applied to the wounds.

**Experiment**	**N**	**Epithelial appendages present on day 30**	**Epithelial appendages present on day 90**
TOPGAL	8	0/8	-
BATGAL	8	0/8	-
LiCl	6	5/6	-
Control	6	0/6	-
PMX-Wnt5A	21	11/14	5/7
PMX-CD5	21	0/14	0/7

At the last time point examined (day 90), 70% of the PMXWnt-5a treated wounds (5/7 mice) contained epithelial cysts and occasional rudimentary hair follicles and sebaceous glands within the healed wound. We saw similar changes throughout the epithelium overlying the wound, but the epithelial findings were most numerous near the wound edge where expression of the CD5 marker and hence Wnt-5a, was most concentrated. Though other labs have observed that activation of β-catenin-dependent signaling in the epidermis resulted in epithelial tumors [8], we did not observe any tumors in the PMXWnt-5a treated mice wounds. The mice remained healthy and thriving 90 days after wounding.

Wnt-5a typically signals via the β-catenin-independent pathway but may also activate the β-catenin-dependent pathway depending on cellular context [16]. Because the epithelial findings after PMXWnt-5a treatment was similar to the epithelial changes with LiCl, we determined whether Wnt-5a was activating the β-catenin-dependent pathway. In a separate group of TOPGAL mice, we applied either PMXWnt-5a retrovirus or Wnt-5a conditioned media topically to the wounds, but did not see any change in β-galactosidase activity compared with the control wounds (data not shown). To confirm that Wnt-5a did not affect the stability of β-catenin in vivo, we excised PMXWnt-5a treated wounds at multiple time points and analyzed free cytosolic β-catenin. Expression of Wnt-5a in the healing wound made no difference in the abundance of free cytosolic β-catenin (Fig. 3j). We also examined the effect of PMXWnt-5a retrovirus on TOPGAL mice and found no difference in β-gal activity compared with controls (data not shown). Prolonged expression of Wnt-5a in vivo likely led to differentiation of the epithelial keratinocytes to a follicular phenotype via activation of β-catenin-independent signaling, though we cannot discount subtle activation of β-catenin-dependent signaling. Thus, we conclude that activation of either Wnt signaling pathway in vivo can lead to morphogenetic changes in the epithelium of adult mammalian wounds; changes that mimic regenerative healing.

## Discussion

Adult mammalian skin is an organ that responds to injury with reestablishment of an epithelial barrier, but does not recapitulate development to reestablish the original epithelial tissue pattern. The healed cutaneous wound lacks the hair follicles, sebaceous and sweat glands and is replaced by scar: a layer of stratified interfollicular epithelium overlying a collagen-rich mesenchymal cell layer. Whereas scar tissue over a small surface area is rarely problematic, the loss of skin over a larger surface area can be functionally devastating and life threatening.

The hair follicle is a naturally regenerating system that undergoes cycles of growth (anagen), regression (catagen) and rest (telogen). The regenerative capability of the hair follicle is lost after skin injury in adult mammals, and the wound is epithelialized with interfollicular epithelium. Regeneration is not seen in most mammalian adult organ systems even though evidence is abound that stem cells or cells capable of restoring the original tissue are present within multiple adult tissues [2,5,17-23]. For regeneration to occur in adult mammalian skin, the cells that repopulate a wound must show responsiveness to the same morphogenetic signals that guide skin development [1,24] and the gradient of morphogenetic signals must be present in the correct environmental context.

In this paper, we demonstrate that the interfollicular epithelial cells that repopulate the wound surface are capable of responding to morphogenetic signals to reform epithelial appendages, either resulting from inhibition of GSK-3β and activation of β-catenin-dependent signaling or the prolonged presence of Wnt-5a arising from the deeper mesenchymal cells. Using either method, we demonstrate the restoration of tissue patterning in the adult mammalian wound epithelium – a feature not normally seen in adult cutaneous wound healing. We controlled for the hair cycle phase that naturally occurs by sex and age-matching the experimental and control groups. Though we could not completely control for the subtle variation, in which one mice is in anagen phase and the other in telogen, the lack of any epithelial appendage formation in the control group suggests this to be a minor variable.

We observed similar regenerative epithelial changes in the wounds after activation of the β-catenin-dependent pathway (LiCL) as well as with PMXWnt-5a, which as best as we could tell, did not appear to activate the β-catenin-dependent pathway. Though we were unable to detect stabilization of β-catenin or increased activity of β-gal in TOPGAL mice treated with PMXWnt-5a retrovirus, we cannot exclude subtle activation of the β-catenin-dependent pathway. Wnt-5a classically signals via the β-catenin-independent pathway and we assume activation of this pathway is involved in the observed regenerative epithelial response, but cannot confirm until more robust assays are developed for the β-catenin-independent pathway. One explanation for our observations is that the prolonged presence of a canonical activator (LiCl) or Wnt-5a non-canonical ligand changed the balance of secondary signaling molecules, making it permissive for epithelial differentiation to occur. Further experiments are needed to determine whether the genes that control epithelial appendage during development were altered, including Sonic Hedgehog [10,25,26] or c-myc [27,28].

Our results showed the development of epithelial cysts in the dermis lined by interfollicular epithelial cells and occasional sebaceous glands. The histology of the observed cysts is similar to that seen in the ?NLef1 transgenic mouse, which developed cysts filled with interfollicular epithelial cells and sebocytes at the base of hair follicles [29]. We did not observe the formation of hair follicles as seen with the β-catenin transgenic mice [8,30]. Although the signaling mechanisms activated in our wound healing model is unclear, it is clear that Wnt signaling can have a profound influence on epithelial cell differentiation not only during development, but also in response to injury.

## Conclusion

The wound epithelium in adult mammals is capable of responding to morphogenic signals from the dermis, as it does in the embryo during hair placode formation. We speculate that the forced expression of a secreted Wnt-5a from the deeper wound resulted in invasiveness [31] of the stratified interfollicular epithelium towards the morphogenic gradient with epithelial appendage formation. Though unclear as to whether both or additional pathways are being activated by LiCl and Wnt-5a, our data establish that the interfollicular epithelial cells in a mammalian adult wound are capable of responding to Wnt signaling with morphogenetic movements mimicking epithelial regeneration. This has important implications in clinical medicine, for the treatment of patients that suffer the consequence of healing large cutaneous wounds without epithelial appendage formation.

## Methods

### Animal wounding model

All animal procedures are in accordance with the *Guide for the Care and Use of Laboratory Animals *and have been approved by the Animal Care Committee of the University of Washington. Either male C57Bl/6J mice (Jackson Labs), male TOPGAL mice (kindly provided by Dr. E. Fuchs) [12] or male BATGAL mice (kindly provided by Dr. S. Piccolo) [13] between 8 – 12 weeks of age were used for the wounding experiments. Mice were anesthetized by intraperitoneal injection of a ketamine and xylazine mixture (15 mg/kg and 1 mg/kg respectively; Phoenix Pharmaceuticals Inc.). The dorsal hair was removed and skin prepared for generation of a standardized 1.5 cm^2 ^full thickness wound (including the panniculus carnosus muscle) on the midback. The wound was covered with a transparent semi-occlusive dressing (Tegaderm, 3M) to prevent desiccation. Manipulation of the wound bed for each experiment is described in subsequent sections.

### β-catenin-dependent Wnt activation

LiCl represses glycogen synthase kinase-3β activity, resulting in accumulation of β-catenin in the cytoplasm and nucleus and activation of the β-catenin-dependent Wnt pathway. Lithium chloride (20 mM, 0.2 ml, n = 14 TOPGAL mice) or control DMEM media (n = 14 TOPGAL mice) was topically applied to the wound under the occlusive dressing, allowing the solution to bathe the wound. Exposure of the wound to LiCl or control media was repeated on a daily basis until wound closure. On days 7, 14, 21, and 30 two mice from each time point and each group were euthanized and their wounds photographed. The photographs were digitized (Nikon Cool Scanner) and analyzed by NIH Image software (v 1.62) to measure the wound size in mm^2^. The excised wound was fixed in 10% formalin for histological analysis.

### Real time PCR

RNA was extracted from the wounds of BATGAL mice at 0, 7, 14, 21 and 28 days (N = 8 mice, 2 per time point) using a liquid nitrogen-cooled mortar and pestle and homogenized in Trizol Reagent (GibcoBRL) [32,33]. RNA was purified further using RNeasy mini columns (Qiagen). cDNA was synthesized using 2 μg of total RNA in a volume of 20 μl. Real time PCR was performed in an ABI Prism 7900 HT instrument using the QIAGEN QuantiTect SYBR Green PCR kit. Primers were designed to correspond to different exons to avoid amplification of genomic DNA. Primer sequences included (all are 5' to 3') Wnt4: CTGGAGAAGTGTGGCTGTGA & GGACGTCCACAAAGGACTGT; Wnt5A: GGCATCAAGGAATGCCAGTA & GTACGTGAAGGCCGTCTCTC; Wnt11: GTAGGGCCTTCGCTGACAT & CGATGGTGTGACTGATGGTG. The annealing temperature for all primer sets was 55°C. GAPDH was used as an endogenous control and the Day 0 (normal skin) sample was used as the calibrator. This experiment was confirmed with TOPGAL mice, whose wounds were harvested on days 0, 3, 7, 14, and 21 (3 mice per time point).

### Retroviral constructs

Full-length Wnt-5a was subcloned into PMXIRES (provided by G. Nolan, Stanford University, CA), a bicistronic retroviral vector containing a non-functional human CD5 cell surface marker [34]. Thus the retroviral construct produces two proteins, the protein of interest and a cell surface non-functional tag, human CD5, to verify the infected cell is producing protein. All retroviral constructs were verified by dideoxy sequencing. Protocol for production of recombinant retrovirus were obtained from the Nolan Lab website [35]. In brief, PMXWnt5a, or control PMXCD5 vector were transfected into Phoenix packaging cells with cytofectine (BioRad Laboratories, CA). After 48 hours, retroviral media was collected and polybrene added to a final concentration of 10 μg/ml. Each lot of retroviral media was tested for infection efficiency by incubating a mouse endothelial cell line (CRL2280; ATCC) for 24 hours, staining with PE-conjugated anti-CD5 antibody and analyzing human CD5 epitope expression by flow cytometry. All retroviral media used in the wounding experiments showed ≥ 35% infectivity in vitro and between 9–17% infectivity in vivo [15].

### Retroviral wounding experiments

In a separate group of C57Bl mice, the wounds were covered with retroviral media containing PMXWnt-5a (36 mice, 6 per time point), or control PMXCD5 (36 mice, 6 per time point). Daily application of retrovirus was performed topically under the clear dressing to cover the entire wound and repeated daily until wound closure. On days 7, 14, 21, 30, 40 and 90, wounds were excised and processed for histology and immunohistochemistry.

### Wound closure rate analysis

Wounds were digitally photographed at the time of generation (Day 0) and again on days 7, 14, 21 and 30, or until wound closure. Wound area was measured using NIH Image.

### Western blot analysis

Tissues were homogenized in RIPA buffer and Protease Inhibitors Cocktail (1 ml/20 g tissue; Sigma) for 5 minutes on ice. PMSF (30 μl/g tissue) was added and lysates incubated on ice for 30 minutes. Lysates were centrifuged and quantified in BCA assays. All SDS-PAGE gels were loaded with 10 μg protein/lane, run and transferred according to standard protocol. Western analysis was performed with anti-β-catenin antibody (Sigma).

### Histology and Immunohistochemistry

Wounds were excised, bisected along the cranial-caudal axis and either frozen in OCT (Tissue-Tek, Sakura) or placed in 10% formalin overnight. Frozen tissues were cut at 10 μm sections, post-fixed in 100% cold acetone, blocked for 1 hour with goat serum and then incubated with a PE-labeled anti-CD5 antibody (BD-Pharmigen, CA) for one hour. Tissues were then counterstained for 5 minutes with DAPI (Molecular Probes, OR) to visualize nuclei. Images were captured with a Nikon Coolpix 995 camera. For tissues fixed in formalin, tissues were embedded, cut and stained with hematoxylin and eosin for further analysis.

## Authors' contributions

CF, BK and AH were responsible for generating the majority of the data, including analysis of the wound healing experiments, RT-PCR, TOPFLASH assay and western blots. LW was primarily responsible for the animal experiments. KS provided the retroviral constructs for Wnt overexpression experiments. RM and FI were the senior investigators who conceived the study and participated in its design and coordination. All authors read and approved the final manuscript.
